# Protocol implementation during the COVID-19 pandemic: experiences from a randomized trial of stress ulcer prophylaxis

**DOI:** 10.1186/s12874-024-02233-2

**Published:** 2024-05-04

**Authors:** Brittany Dennis, Adam Deane, François Lauzier, Nicole Zytaruk, Miranda Hardie, Naomi Hammond, Simon Finfer, Yaseen Arabi, John Marshall, Lois Saunders, Diane Heels-Ansdell, John Myburgh, Serena Knowles, John Muscedere, Marlies Ostermann, Dorrilyn Rajbhandari, Shane English, Karlo Matic, Bala Venkatesh, Abdulrahman Al Fares, Gordon Guyatt, Waleed Alhazzani, Hassan Mumtaz, Alexis Poole, Feng Xie, Lehana Thabane, Richard Hall, Deborah Cook

**Affiliations:** 1 Department of Medicine, McMaster University, Hamilton, Ontario Canada; 2 Department of Critical Care Or Medicine, Department of Critical Care Medicine, Melbourne Medical School, University of Melbourne, Parkville, Victoria Australia; 3 Departments of Anesthesiology, Medicine and Critical Care Medicine, Université Laval, Québec, Canada; 4 Department of Health Research Methods, Evidence, and Impact, McMaster University, Hamilton, Ontario Canada; 5 Division of Critical Care, Research Institute, St. Joseph’s Healthcare Hamilton, Hamilton, Ontario Canada; 6 Critical Care Program, Faculty of Medicine, The George Institute for Global Health, University of New South Wales, Sydney, NSW Australia; 7 Intensive Care Department, Ministry of the National Guard-Health Affairs, Riyadh, Kingdom of Saudi Arabia; 8 King Saud Bin Abdulaziz University for Health Sciences, Riyadh, Kingdom of Saudi Arabia; 9 King Abdullah International Medical Research Center, Riyadh, Kingdom of Saudi Arabia; 10 Interdepartmental Division of Critical Care, University of Toronto, Toronto, Ontario Canada; 11 Intensive Care Unit, St. George Hospital, Sydney, Australia; 12 Department of Critical Care Medicine, Queen’s University, Kingston, Ontario Canada; 13 Department of Critical Care, King’s College London, Thomas’ Hospital, Guy’s & St, London, UK; 14 Department of Medicine, University of Ottawa, Ottawa, Ontario Canada; 15 Clinical Epidemiology Program, Ottawa Hospital Research Institute, Ottawa, Ontario Canada; 16 Departments of Anesthesia, Critical Care Medicine, and Pain Medicine, Al-Amiri Center for Respiratory and Cardiac Failure, Al-Amiri Hospital, Ministry of Health, Kuwait Extracorporeal Life Support Program, Ministry of Health, Kuwait City, Kuwait; 17 Department of Critical Care, Maroof Hospital, Islamabad, Pakistan; 18 School of Public Health and Preventive Medicine, Monash University, Melbourne, Victoria Australia; 19 Discipline of Acute Care Medicine, University of Adelaide, Adelaide, South Australia Australia; 20 Centre for Research Excellence in Translating Nutrition Science to Good Health, University of Adelaide, Adelaide, South Australia Australia; 21 Biostatistics Unit, St. Joseph’s Healthcare Hamilton, Hamilton, Ontario Canada; 22 Departments of Anesthesia, Critical Care and Pharmacology, Dalhousie University, Halifax, NS Canada

**Keywords:** Randomization, Critically ill, COVID-19 Pandemic, Hospital transfers, Stress ulceration, Gastrointestinal bleeding, Ventilator-associated pneumonia

## Abstract

**Background:**

During the COVID-19 pandemic, many intensive care units (ICUs) halted research to focus on COVID-19-specific studies.

**Objective:**

To describe the conduct of an international randomized trial of stress ulcer prophylaxis (Re-Evaluating the Inhibition of Stress Erosions in the ICU [REVISE]) during the pandemic, addressing enrolment patterns, center engagement, informed consent processes, data collection, a COVID-specific substudy, patient transfers, and data monitoring.

**Methods:**

REVISE is a randomized trial among mechanically ventilated patients, comparing pantoprazole 40 mg IV to placebo on the primary *efficacy* outcome of clinically important upper gastrointestinal bleeding and the primary *safety* outcome of 90-day mortality. We documented protocol implementation status from March 11th 2020-August 30th 2022.

**Results:**

The Steering Committee did not change the scientific protocol. From the first enrolment on July 9th 2019 to March 10th 2020 (8 months preceding the pandemic), 267 patients were enrolled in 18 centers. From March 11th 2020-August 30th 2022 (30 months thereafter), 41 new centers joined; 59 were participating by August 30th 2022 which enrolled 2961 patients. During a total of 1235 enrolment-months in the pandemic phase, enrolment paused for 106 (8.6%) months in aggregate (median 3 months, interquartile range 2;6). Protocol implementation involved a shift from the a priori consent model pre-pandemic (188, 58.8%) to the *consent to continue* model (1615, 54.1%, *p* < 0.01). In one new center, an *opt-out* model was approved. The informed consent rate increased slightly (80.7% to 85.0%, *p* = 0.05). Telephone consent encounters increased (16.6% to 68.2%, *p* < 0.001). Surge capacity necessitated intra-institutional transfers; receiving centers continued protocol implementation whenever possible. We developed a nested COVID-19 substudy. The Methods Centers continued central statistical monitoring of trial metrics. Site monitoring was initially remote, then in-person when restrictions lifted.

**Conclusion:**

Protocol implementation adaptations during the pandemic included a shift in the consent model, a sustained high consent rate, and launch of a COVID-19 substudy. Recruitment increased as new centers joined, patient transfers were optimized, and monitoring methods were adapted.

## Introduction

In March 2020 when the SARS-CoV-2 pandemic was declared, most research for critically ill patients was halted to prioritize investigations on the diagnosis, prognosis and treatment of patients with SARS-CoV-2. The pandemic jeopardized standard research processes in many circumstances, resulting in consent withdrawal, impaired protocol fidelity, and incomplete data collection [1, 2]. Restricted hospital presence of non-essential staff and citizen lock-downs required some research personnel to work remotely, limiting in-person contact with study participants and their families [3]. Additional consequences included postponed procedures to preserve personal protective equipment, supply chain interruptions for investigational drugs and devices, and premature study closure [3–8]. During the height of the pandemic, research in the intensive care unit (ICU) setting faced several additional challenges including research staff redeployment to the bedside, new requirements for data monitoring, and more stringent infection control procedures [4, 5, 9].

Rapidly launched clinical trials focused on SARS-CoV-2 generated key pharmacotherapeutic information to guide practice around the world [10–16]. Pre-existing and newly developed adaptive platform trials allowed simultaneous comparison of multiple intervention groups against a single control group, thereby facilitating rapid therapeutic discoveries [17]. Greater use of expedited and centralized research ethics committee reviews, and alternate consent models as suggested following the 2009 H1N1 pandemic were adopted [18]. The global response of clinical trials during the pandemic was largely reactive rather than anticipatory [19], emphasizing areas for improvement in the implementation of research during a state of emergency. Investigators have generated suggestions to facilitate the conduct of clinical trials during these timeses [20]. Understanding the impact of the pandemic on ongoing clinical trials may also help to inform future contingency plans for the next health crisis.

Operational throughout the pandemic, the Re-Evaluating the Inhibition of Stress Erosions (REVISE) Trial is an international randomized trial of stress ulcer prophylaxis comparing a proton pump inhibitor (PPI) versus placebo for invasively ventilated patients to prevent upper gastrointestinal (GI) bleeding [NCT03374800] [21]. In addition to challenges of trial conduct, investigators needed to rapidly evaluate emerging data about the safety of PPIs in patients with COVID-19 and consider whether they were eligible for REVISE. Observational studies about SARS-CoV-2 suggested that short-term PPI use was associated with increased risk of admission to the ICU, use of mechanical ventilation, or death [22]. Analysis of 2,164 hospitalized patients with COVID-19 suggested that PPI exposure was associated with higher mortality, but not after adjusting for comorbidities [23]. A signal for increased risk of death from COVID-19 infection in those receiving PPIs (risk ratio 1.7, 95% CI 1.02–2.9) was also found in a pooled analysis of 6 studies enrolling 5,884 patients [24]. In practice, in light of high-dose corticosteroid treatment [10], many mechanically ventilated patients with COVID-19 receive acid suppression [25]. Given the absence of randomized trial data directly relevant to this vulnerable population, patients with SARS-CoV-2 were considered eligible for the REVISE Trial.

The objective of this report is to describe REVISE protocol implementation before and during the first four pandemic waves, addressing enrolment patterns, center engagement, informed consent processes, data collection, a COVID-specific substudy, patient transfers, and data monitoring.

## Methods

### Overview of REVISE methods

REVISE is an international randomized stratified, concealed, blinded, parallel group trial of adults undergoing invasive mechanical ventilation who are randomized to receive either pantoprazole 40 mg intravenously or placebo. The primary *efficacy* outcome is clinically important upper gastrointestinal bleeding, and the primary *safety* outcome is 90-day mortality. Secondary outcomes include ventilator-associated pneumonia (VAP), *Clostridioides difficile*infection, patient important gastrointestinal bleeding and other endpoints [26]. Eligible patients are invasively mechanically ventilated and expected to be so for the next 48 h (or the day after tomorrow). Inclusion and exclusion criteria are presented in Table 1.

**Table 1:** Summary of the trial eligibility criteria

**Inclusion criterion**
Adults ≥ 18 years old projected to received invasive mechanical ventilation for ≥ 48 h according to the treating physician
**Exclusion criteria**
1. Already received invasive mechanical ventilation ≥ 72 h during this hospital admission 2. Acid suppression for active GI bleeding or high risk of bleeding (e.g., current bleeding, peptic ulcer bleeding within 8 weeks, recent severe esophagitis, Barrett's esophagus, Zollinger-Ellison syndrome); [dyspepsia or gastroesophageal reflux is not an exclusion criterion] 3. Acid suppression in the ICU for > 1 daily dose equivalent of a PPI or H2RA 4. Dual antiplatelet therapy 5. Combined antiplatelet and therapeutic anticoagulation 6. Pantoprazole contraindication per local product information 7. Palliative care or anticipated withdrawal of advanced life support 8. Pregnancy 9. Previous enrolment in REVISE, or a related trial, or trial for which co-enrolment is prohibited 10. Patient, proxy or physician declines

This table shows the eligibility criteria for this COVID-19 Substudy, reflecting inclusion and exclusion criteria for the REVISE trial

*ICU* intensive care unit, *PPI* proton pump inhibitor, *H2RA* histamine-2-receptor antagonists

Research staff screen patients for eligibility; when confirmed, the protocol allows either a priori informed consent before randomization or informed *consent to continue* after randomization (e.g., randomized followed by consent, or *deferred consent*in some jurisdictions). Research pharmacists or other unblinded staff randomize patients to receive 40 mg pantoprazole or identical placebo daily while patients are undergoing invasive ventilation. All other stakeholders are blinded. Research staff collect daily data, study drug adherence and trial outcomes for up to 90 days. The target sample size is 4,800 patients [26].

### Approach to data collection

To document the dynamic influence of the pandemic on trial recruitment at monthly Methods Center meetings, we used both retrospective and prospective data collection methods. From March 11th 2020 to August 30th 2022, research staff in participating centers prospectively liaised with the Methods Center to share the status of the trial whether enrolment was paused or pursued each month. Retrospectively, Methods Center staff validated the status of the trial with participating centers, documenting monthly whether a) all ICU research was paused, b) only COVID-19-specific research was ongoing, c) whether both COVID-19-specific and other research was ongoing, or d) whether launching REVISE was directly delayed due to the pandemic. For patient transfers to non-participating sites, research staff ensured accurate contact information and arranged follow-up for 90-day mortality status whenever possible.

### Statistical analysis

We define the pre-pandemic time-frame from the first patient enrolment on July 9th 2019 to March 10th 2020 inclusive (8 months). As per the global pandemic declaration by the World Health Organization on March 11th 2020 [27], we considered the pandemic period from March 11th 2020-August 31st 2022 (30 months). We analyzed types of consent encounters, the informed consent rate, center participation, and patient recruitment over these two periods.

To analyze center participation, we defined ‘pre-pandemic sites’ as those in which patients were enrolled, or in which there was a priori patient or substitute decision-maker decline to consent, from July 9th 2019 up to and including March 10th 2020. Sites enrolling patients between March 11th 2020 and August 31st 2022 also included pre-pandemic sites.

We compared the consent models and consent rate in REVISE in the pre-pandemic period to that observed in the pandemic period using Fisher’s Exact test. A sum of candidate enrolment-months, defined as months during which centers were open for trial participation, was calculated by summing all active screening months; this was used as the denominator to calculate the percentage of paused months per center during the study period.

We report data using descriptive statistics as counts and percentages, mean and standard deviation or median and interquartile range (IQR), as appropriate.

### Ethics

REVISE was approved by relevant research ethics committees as required in each jurisdiction.

## Results

### Patient enrolment

In total, 2961 patients were enrolled into REVISE from the trial launch of July 9th 2019 to August 31st 2022. We included 2952 patients in this analysis; 9 were excluded (5 due to missing consent information, 2 because of randomization errors, and 2 awaiting data release approval from local governance organizations).

In the 8-month pre-pandemic period (July 9th 2019 to March 10th 2020), 267 (9.0%) patients were randomized, and in the following 30-month period (March 11th 2020 to August 31st 2022), 2685 (91.0%) patients were randomized.

The overall consent rate pre-pandemic was 242 of 300 consent encounters (80.7%) compared to 2331 of 2743 consent encounters (85.0%) thereafter (*p* = 0.05).

### Center engagement

In the 8-month pre-pandemic period (July 9th 2019 to March 10th 2020), 18 centers had started REVISE; in the following 30-month period (Mar 11th 2020 to August 31st 2022), 41 additional centers started REVISE. Therefore, a total of 59 centers participated in REVISE trial between July 9th 2019 and August 31st 2022. During this period, 22 sites required a pause in recruitment. Pauses occurred for multiple concurrent reasons, including mandates from hospitals or universities to halt all research, directives to prioritize COVID-19 related studies, and to redeploy research staff to clinical care.

**Fig. 1:**
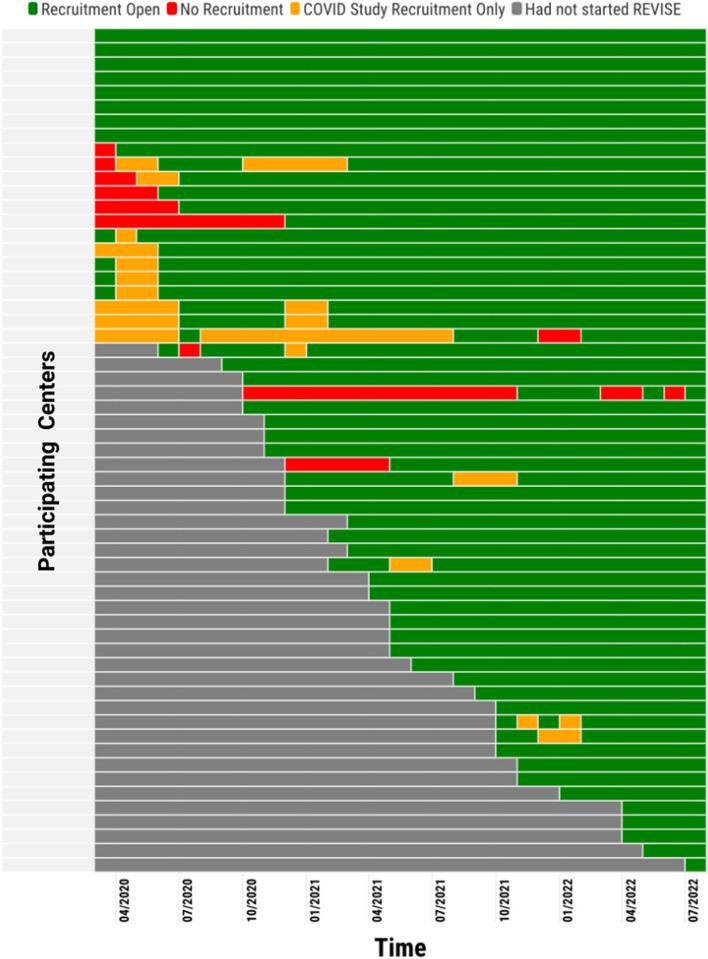
Enrollment status of revise centers during the COVID-19 Pandemic**.** This figure shows recruitment status across participating REVISE centers from March 11th 2020 to August 31st 2022. The X axis refers to time (months in quarters) and each row in the Y axis represents a participating center

Fig. 1 presents a summary of REVISE center enrollment status. Enrolment paused for the shortest possible periods as jurisdictionally admissible in each site. All research (including COVID-19) paused in 10 (16.9%) centers, and 17 (28.8%) centers had periods in which investigations were directed at COVID-19 during this study period. During the 30-month period after pandemic declaration, across 59 centers, there were a total of 1235 candidate enrolment months (median 22 months/center, IQR 15;30 months]. During these 1235 months, enrolment was paused for a total of 106 (8.6%) months due to the pandemic. The median duration of paused recruitment months per center was 3 months (IQR: 2;6 months). Participating centers during this 30-month period continued active recruitment for a median of 19 months [IQR: 12; 27 months].

### Informed consent

Informed consent models approved for REVISE pre-pandemic included a priori consent and *consent to continue*. During the 30-month pandemic period, hospital visiting restrictions limited in-person consent encounters with families. We documented a shift from primary use of the a priori consent model pre-pandemic (188, 58.8% of consent encounters) to the *consent to continue* model (1615, 54.1% of consent encounters), *p* < 0.01, during the pandemic. In one center joining the trial during the pandemic, the *opt out* model was approved (patients or families can opt out of the study following randomization). Consenting scenarios employed across pre-pandemic and pandemic timelines are summarized in Table 2.

**Table 2:** Revise consent scenarios

	**Pre-Pandemic** ***N*** = 320	**Pandemic** ***N*** = 3022	**Total** ***N*** = 3342
Patient consented	45 (14.1)	579 (19.2)	624 (18.7)
Substitute Decision Maker consented	197 (61.6)	1744 (57.7)	1941 (58.1)
Other consented	0 (0.0)	8 (0.3)	8 (0.2)
Opt out Model was used (no Patient or Substitute Decision Maker opted out)	Not applicable	36 (1.2)	36 (1.1)
Patient declined	2 (0.6)	13 (0.4)	15 (0.4)
Substitute Decision Maker declined	56 (17.5)	399 (13.2)	455 (13.6)
No consent encounter – Patient lacked capacity to consent, no Substitute Decision Maker	7 (2.2)	86 (2.8)	93 (2.8)
No consent encounter – Patient died, never had capacity to consent, no Substitute Decision Maker	10 (3.1)	154 (5.1)	164 (4.9)
No consent encounter – Other circumstances	3 (0.9)	3 (0.1)	6 (0.2)

This table shows consent scenarios pre-pandemic and during the pandemic. We defined pre-pandemic as up to and including March 10th, 2020. We defined pandemic as from March 11th up to August 31st, 2022. This analysis includes 2952 patients enrolled, plus 390 patients with *a* priori consent declined for a total of 3342 patients

By August 31st 2022, 52 of 57 participating centers (91.2%) allowed for telephone consent. Among sites that initiated enrollment from the pre-pandemic period onwards, 41 of 247 (16.6%) of consent encounters occurred by telephone prior to the pandemic, which increased after the pandemic was declared to 850 of 1246 (68.2%), *p* < 0.001.

### Data collection

In some centers, data collection methods were modified whereby research staff received secure remote access to medical records for screening, data collection and data entry. Otherwise, data collection proceeded as usual.

**Fig. 2:**
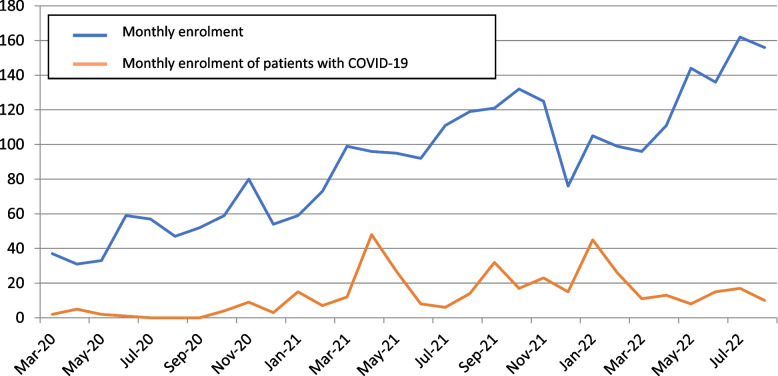
Revise enrolment (March 2020-August 2022).This figure shows recruitment per month and enrolment of patients with COVID-19 across 61 REVISE centers from March 11th 2020 to August 31st 2022. The X axis refers to months and each row in the Y axis refers to number of enrolled patients per month

We adapted REVISE case report forms to document SARS-CoV-2 status and created a new one-page form to document vaccination status, biomarkers, venous thromboembolism, tracheostomy timing and COVID-19 treatments. From March 11th 2020 to August 31st 2022, we documented that the proportion of REVISE patients with SARS-CoV-2 during their index hospital admission increased from none to 396 of 2657 (14.9%) of patients (Fig. 2).

### COVID-19 cohort substudy

We obtained peer-reviewed funding to further characterize patients with SARS-CoV-2 in REVISE. The objectives are to 1) characterize patients with COVID-19 in terms of demographics, biomarkers, rates of venous thromboembolism, duration of mechanical ventilation, ICU and hospital stay, tracheostomy timing and mortality, 2) evaluate the impact of COVID-19 on clinically important GI bleeding, 90-day mortality, and other outcomes compared to a propensity-matched non-COVID-19 cohort; and finally 3) explore the PPI treatment effect on clinically important GI bleeding, 90-day mortality and other outcomes in patients with and without COVID-19 [NCT05715567].

### Patient transfers

In total, 174 (5.9%) patients were transferred to other sites during this study time-frame of July 9th 2019 to August 31st 2022; only a minority of patients (14, 8.0%) transferred for regional referrals or other reasons. Among the 174 transferred patients, 43 patients were transferred only to a site participating in REVISE, 128 were transferred only to a non-participating site, and 3 patients were transferred twice to both a REVISE site and a non-REVISE site.

During the pandemic, the number of patients with COVID-19 surpassed hospital surge capacity in many jurisdictions. To pre-empt intensified triaging of critical care services, many critically ill patients were transferred to other centers, regardless of COVID-19 status or enrolment in REVISE. If an enrolled patient was transferred to another center, research staff from the sending hospital notified the REVISE Methods Center. If the receiving hospital was a participating REVISE site, the Methods Center staff notified the receiving hospital’s research personnel of the incoming patient, so study drug and data collection could continue if possible. The sending hospital’s research pharmacist or unblinded staff preparing study drug also notified the receiving hospital’s research pharmacist or unblinded study staff to ensure that allocated blinded study drug could continue if possible. If the receiving hospital was not participating in REVISE, research personnel sought the 90-day mortality outcome per protocol.

### Data monitoring

The Methods Centers continued data validation throughout this study period, involving at least 3 stages of chart review. Site-specific blinded source documents for gastrointestinal bleeding events continued to be collated for central adjudication. Ventilator-associated pneumonia events continued to be classified and *Clostridioides difficile* infection severity continued to be verified.

Central statistical monitoring was maintained per protocol, including quarterly assessment of trial management metrics. The data safety and monitoring committee reviewed blinded interim mortality data at the 25% recruitment mark, and full blinded interim analysis results at the 50% recruitment mark, each time suggesting to continue the trial.

Initially, site-specific data monitoring occurred remotely due to pandemic restrictions. On-site monitoring began as travel bans were lifted and Methods Center teams were granted access to site medical records.

## Discussion

Although the REVISE trial required no scientific protocol modifications during the pandemic, trial implementation adaptations helped to preserve methodologic and ethical integrity. We analyzed enrolment patterns, center engagement, and informed consent processes. We launched a COVID-19-specific substudy and optimized patient transfers during the pandemic. The Methods Centers continued central statistical monitoring, as well as validation, adjudication, classification and verification of morbidity outcomes, and initiated remote, followed by in-person, site-specific data monitoring.

Due to restricted in-hospital family presence, we observed a significant shift from the a priori consent model toward the previously approved *consent to continue* model in this trial; in one center which launched during the pandemic, an *opt-out*model was approved. The challenges of obtaining informed consent were amplified by rapid deterioration of patients with SARS-CoV-2, lack of family presence at the bedside, inability to reach family within the consent window, and uncertainty about the potential for new therapies [28]. Many adaptations have arisen including waived consent models and increased use of telephone consent, which are often employed in low-risk observational studies or registries during non-pandemic times. Approaches such as consent to continue (e.g., deferred consent), two-physician consent, delayed or waived wet ink signature confirmation have also been described in a systematic review to inform pandemic preparedness [29]. Some alternative consent models have been adopted and considered broadly acceptable to clinicians, patients’ and families when surveyed post-enrolment [30, 31]. Ultimately, these consent adaptations need to honour the ethical principles of beneficence, nonmaleficence, autonomy, and justice during times of global crisis [5].

Trial consent rates vary for myriad reasons, including the context, conditions and protocol particulars. The informed consent rate for REVISE was sustained with a small non-significant shift during the pandemic (80% to 85%). This may reflect testing an intervention that is not directed at, but relevant to, patients with COVID-19. In a single center, multi-study analysis, a significantly lower consent rate was documented for COVID-19-specific trials (78%) compared to other trials (90%), although patients with COVID-19 were significantly more likely to be co-enrolled in 2 or more studies (38%) compared to other patients (16%) [4]. During the second pandemic wave, one multi-center randomized trial documented a doubling of the consent refusal rate from 18 to 35% between the third and fourth quarter of 2021 [32, 33]. Hesitancy to participate in COVID-specific trials was hypothesized to relate to a high proportion of unvaccinated hospitalized adults who used alternative information sources and were skeptical about preventive and therapeutic interventions [32, 34]. Strategies developed in response to the decline in research participation in some studies during the pandemic have included enhanced research staff training on relating to vulnerable and vaccine-hesitant populations, and emphasizing empathetic education on the consequences of COVID-19 [32].

For some trials during the pandemic, virtual-based recruitment methods were used where not previously operational, which many patients support in the context of an active pandemic [35]. In the REVISE trial, we found that telephone consent encounters, already incorporated as necessary in many centers, were significantly more common during the pandemic. Outside the ICU setting, reports have emerged about successful transitions to virtual recruitment and follow-up [36] and intervention delivery at home where possible [37]. For REVISE, virtual contact, if necessary, was already protocolized in the form of telephone contact to ascertain 90-day vital status; this needed no modification. However, new virtual data monitoring processes were developed and implemented early in the pandemic.

Most centers paused enrolment in REVISE for variable periods to prioritize COVID-19-related studies, and some centers previously planning to start the trial understandably delayed the launch. Overwhelming hospital surge capacity necessitated patient transfers to other health jurisdictions to expedite access to care during the height of the pandemic. The REVISE Methods Centers built in efficiencies which included enhanced surveillance of participant movement between sites, collaboration across participating centers to maintain blinding, and multi-directional communication among research personnel across sites and Methods Center staff to maximize continued participation and follow-up. In this study period, 5.9% of patients were transferred to other hospitals (1.6% to other participating REVISE centers). These findings are consistent with a recent trial evaluating probiotics during critical illness, in which 6.5% of patients were transferred to other hospitals (1.5% to other participating trial centers) (Unpublished data, PROSPECT Trial) [38].

The unclear impact of PPIs in COVID-19 patients led to the development of a nested COVID substudy to characterize high-risk patients with SARS-CoV-2. This required modifications to existing case report forms and a new COVID-specific form. Addressing the impacts of COVID-19 within the context of ongoing pre-pandemic studies has occurred in many settings, to evaluate relevant new research questions, and to facilitate ongoing studies in the midst of constraints [39, 40]. The substudy we designed highlights the utility of embedded designs to describe, detect, and interpret the effect of COVID-19 in the context of an ongoing randomized trial [41].

Some aspects of clinical research during a pandemic need to be approached differently from research undertaken under non-emergent circumstances, conducting investigations as efficiently as possible, focused on optimal processes and outcomes for the individual participant, while balancing maximal societal benefit [5]. Ideally, studies designed and conducted during a pandemic (whether COVID-related or not) should be held to the same high methodologic standards as during other times. The pandemic catalyzed diverse risk-mitigating strategies to ensure participant retention and implementation fidelity in the face of unintended interruptions [37]. For example, in a randomized trial of in-bed cycling for critically ill patients, recruitment was paused and modifications to work-flow were instituted such as remote outcome assessments, until recruitment restarted as soon as possible to allow the interim analysis to proceed [42]. Some trials continued through the pandemic and stopped early [43], transparently reporting pandemic circumstances and adaptations [44].

Limitations of this study include report of a single trial experience. Although consent models, consent rates, and enrolment numbers were collected prospectively, active screening status at each center was also retrospectively verified and recall bias cannot be excluded. It was infeasible to track dynamic institutional directives and regional public health mandates at all participating centers, or whether it was ethics committees, hospital administrators, ICU managers, researchers or a combination of stakeholders who made decisions to pause or pursue enrolment. Our analysis does not include time to ethics approval or contract execution for newly participating centers.

Strengths of this study include site-reported data about research processes during various phases of the pandemic affecting this trial. Harnessing suggestions to launch research focused on COVID-19 in ICUs around the world, and to continue non-COVID-19-specific studies as jurisdictionally admissable [5] facilitated ongoing trial conduct. In addition, the REVISE research question is relevant to patients with and without SARS-CoV-2. In this report, we describe several protocol implementation adaptations during a 38-month period in 59 international centers.

## Conclusion

The onset of the COVID-19 pandemic led to major shifts in the prioritization, design and implementation of research. Broader informed consent processes, recruitment tracking, facilitated patient transfers, monitoring adaptations, and enriched data collection related to COVID-19 helped to advance the REVISE trial through to 2023 without sacrificing methodological fidelity. Understanding the diverse impacts of the pandemic and describing the adaptations made to randomized trials for critically ill patients can inform contingency plans in anticipation of other major global events with the potential to impact clinical research in this setting. The pandemic, while devastating, has ushered in new ways to conduct trials which may enhance trial efficiency in the future.

## Data Availability

Following the publication of REVISE, the dataset will be used for secondary observational studies addressing additional hypothesis-driven questions (e.g., predictors of gastrointestinal bleeding). Access by REVISE investigators will follow a submitted rationale, analysis plan and approval by the Management Committee. Requests for access to the dataset by external investigators will be considered following a submitted rationale, analysis plan and approval by the Management Committee and research ethics boards as relevant. Requirements will be stipulated in a pre-specified data sharing agreement. Only de-identified data will be provided and will be transferred via a secure web portal.
